# Supplementary material for: A new dimension for magnetosensitive e-skins: active matrix integrated micro-origami sensor arrays
Source: Nat Commun. 2022 Apr 19;13:2121. doi: 10.1038/s41467-022-29802-7 (PMC9018910; doi:10.1038/s41467-022-29802-7)
Supplement: Supplementary file 1 — Supplementary Information File [file 41467_2022_29802_MOESM1_ESM.pdf]

Supplementary Information

A new dimension for magnetosensitive e-skins: Active matrix integrated micro-origami sensor arrays

Becker et al.

Supplementary Figures

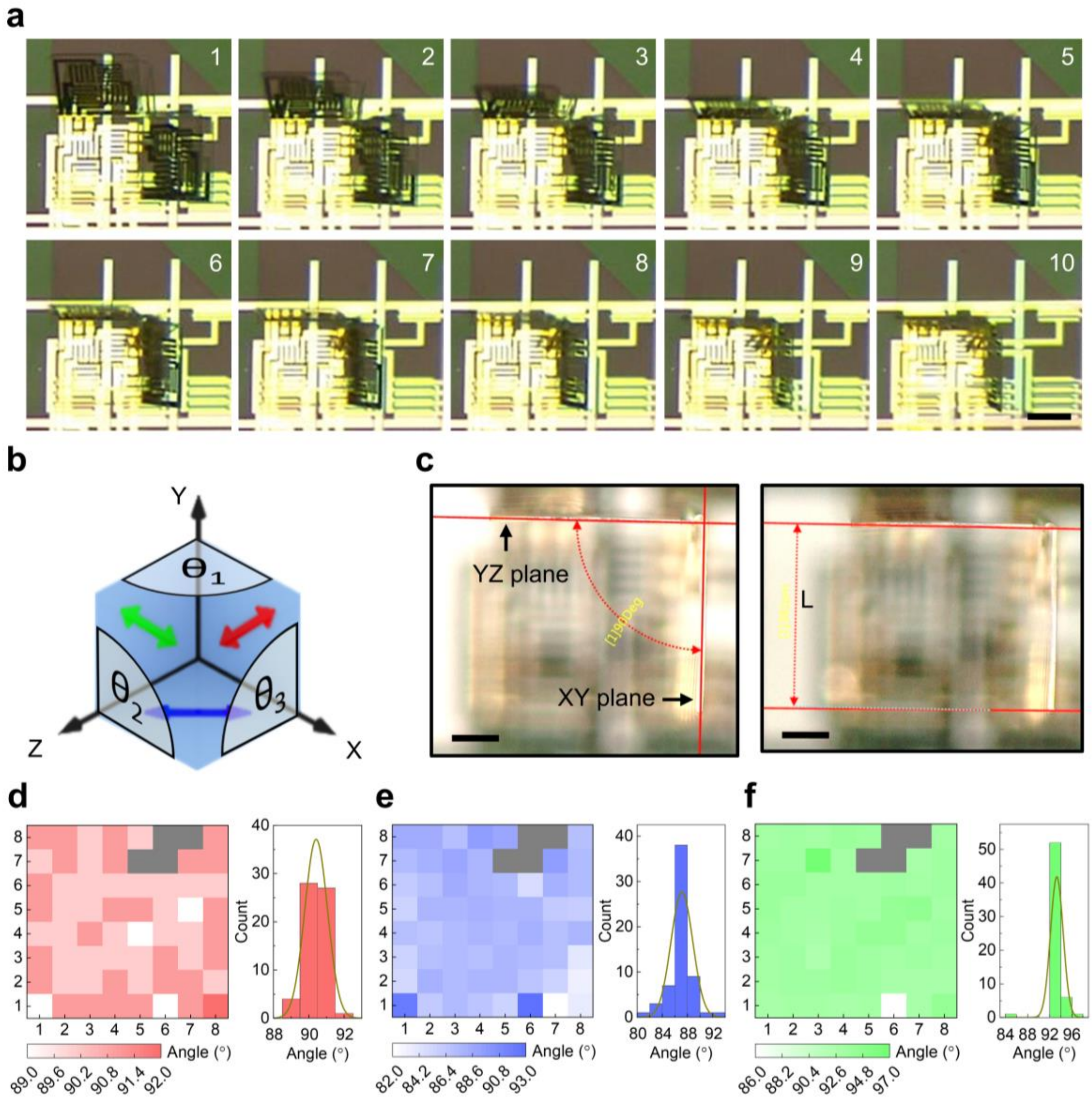

Supplementary Figure 1 | Self-folding process and distributions of the folding angles. a, Self-folding process of a single pixel. Image series showing the self-folding process of a single pixel. Scale bar, 200 µm. **b**, Illustration of the folding angles between different planes of the cubes. **c**, Top view micrographs of a folded sensor pixel showing the measurements of the angle between YZ and XY planes, and the projected distance between the edges of YZ and XZ planes. Scale bars, 100 µm. **d**-**f**, Spatial and statistical distributions of the folding angles θ_1, θ_2 and θ_3. The failed pixels are marked by gray color.

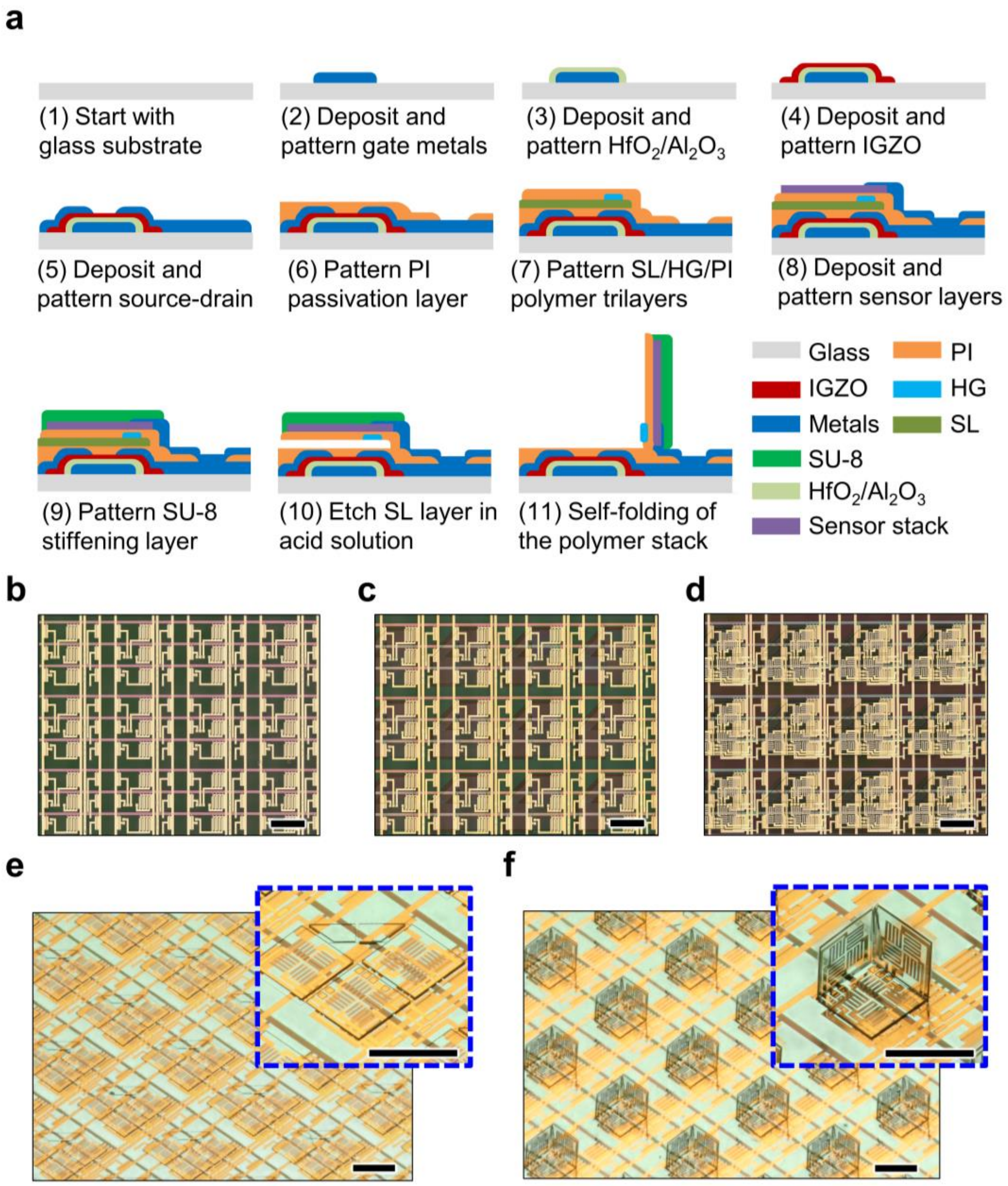

Supplementary Figure 2 | Fabrication process flow and corresponding images of the IMOS device. a, Fabrication process flow of the device. The fabrication process contains three main parts: a-IGZO active matrix backplane fabrication, which includes step (1) to (6); Fabrication of self-foldable polymer stack with embedded electronics, which includes step (7) to (9); Self-folding process which includes step (10) and (11). **b**, Image of a pixel array in the a-IGZO active matrix backplane. **c**, Image of a pixel array of the backplane with SL/HG/PI polymer stack on top of it. **d**, Image of a pixel array of the as-fabricated device in the planar state. **e**, **f**, Side-view images of the device in the planar and folded states respectively. Insets are single pixels in the planar and folded states. Scale bars, 500 µm.

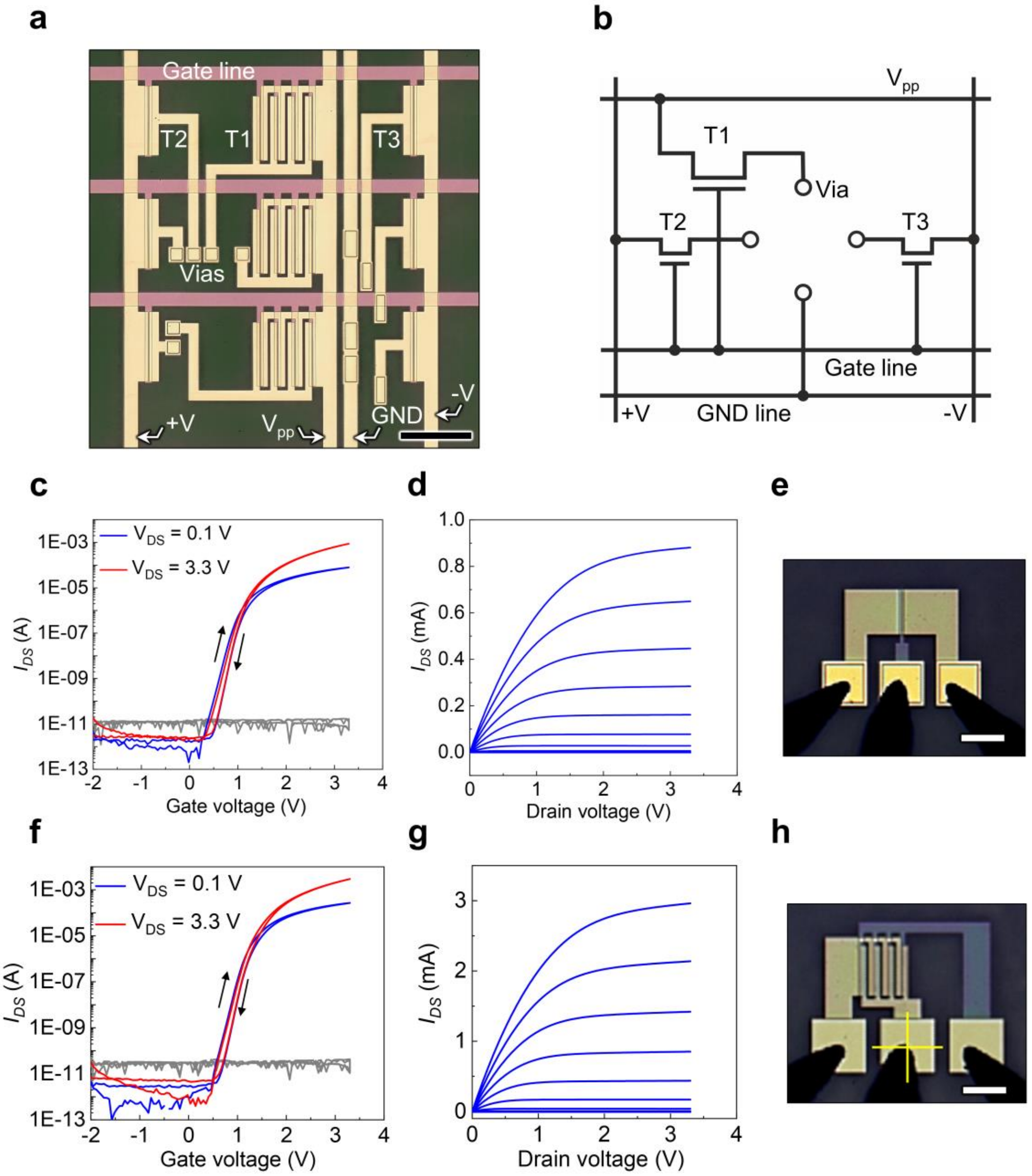

Supplementary Figure 3 | a-IGZO active matrix backplane. a, Image of a single pixel in the active matrix backplane which contains three subpixels to drive the sensors on different sensing planes. **b**, Circuit diagram for a subpixel. Each subpixel contains two rTFTs (T2 and T3, L = 3 µm, W = 200 µm) and one dTFT (T1). The dTFT is composed of four TFTs with the same size as the rTFTs in parallel. All the TFTs in one subpixel share the same gate line thus can be synchronously switched on or off. **c**, **f**, Typical transfer characteristics at V_{GS} from -2 V to 3.3 V for the rTFTs and dTFTs respectively. The leakage currents are plotted with gray lines. **d**, **g**, Corresponding output characteristics for the rTFTs and dTFTs at V_{GS} from -0.3 V to 3.3 V in 0.3 V steps. **e**, **h**, Images of the test structures for rTFT and dTFT. Scale bars, 200 µm.

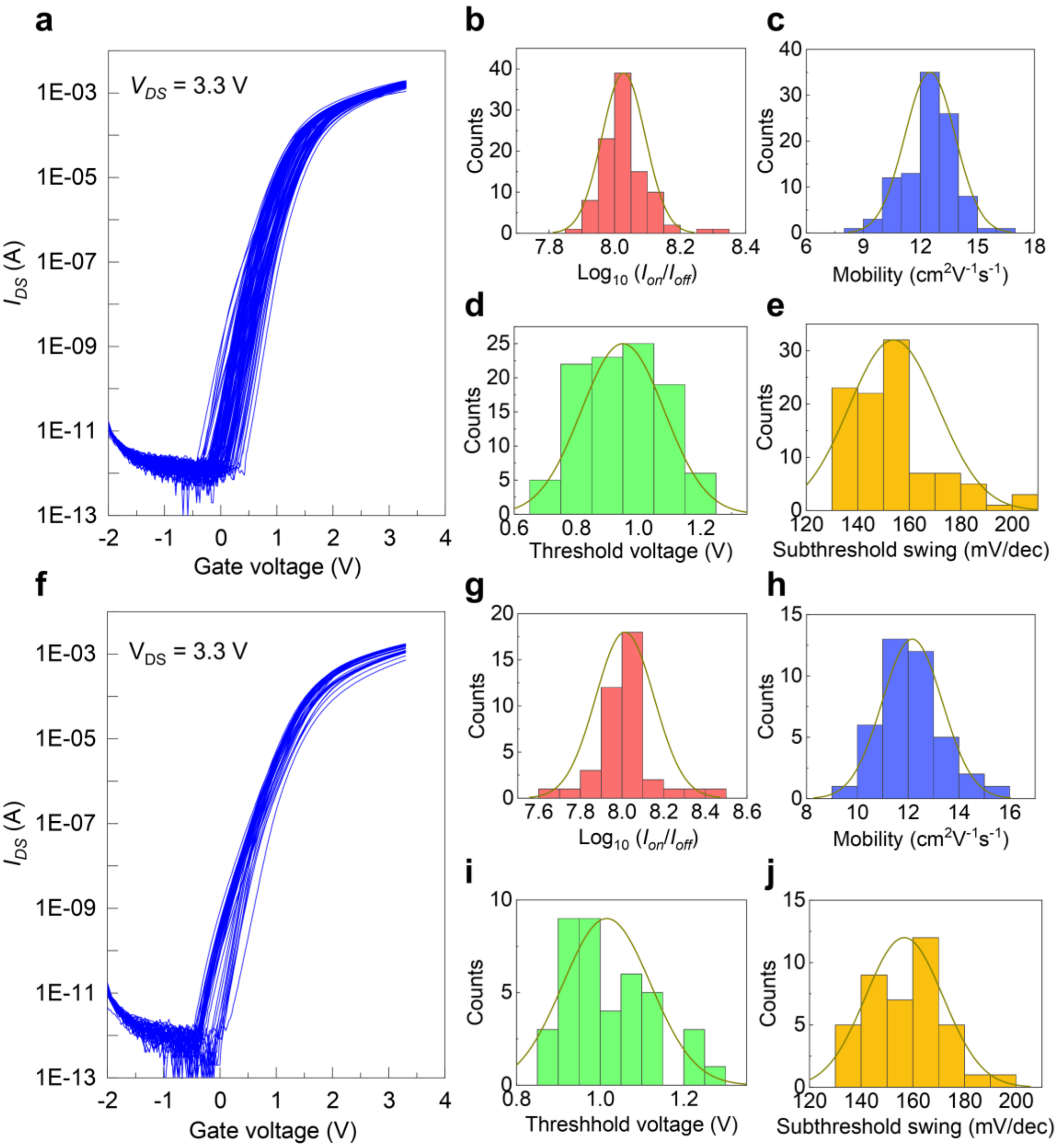

Supplementary Figure 4 | Statistics of the electrical performance for the a-IGZO TFTs before and after self-folding process. a, Transfer characteristics of 100 TFTs tested before the self-folding process. **b**-**e**, Histograms of the on/off ratio, μ, V_{th} and SS of the 100 a-IGZO TFTs showing their statistical distributions. Before the self-folding process, the on/off ratio is (1.08 ± 0.18) × 10^8, the V_{th} is (0.95 ± 0.13) V, the SS is (154 ± 17) mV/dec, and the μ is (12.51 ± 1.32) $cm^2V^{-1}s^{-1}$. **f**, Transfer characteristics of 40 TFTs tested after the self-folding process. **g**-**j**, Histograms of the on/off ratio, μ, V_{th} and SS of the 40 a-IGZO TFTs. After the self-folding process, the on/off ratio is (1.09 ± 0.42) × 10^8, the V_{th} is (1.02 ± 0.10) V, the SS is (157 ± 15) mV/dec, and the μ is (12.16 ± 1.19) $cm^2V^{-1}s^{-1}$. Therefore, the self-folding process has neglectable influence on the electrical performance of the a-IGZO TFTs.

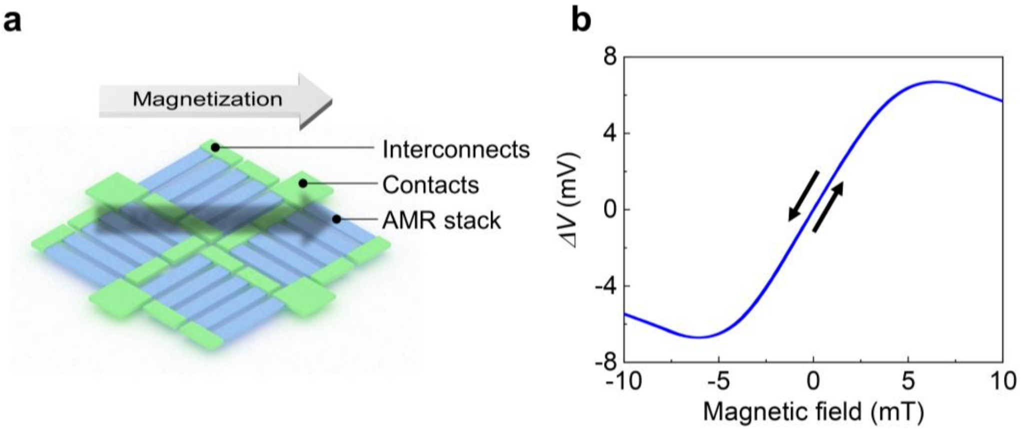

Supplementary Figure 5 | Structure and magnetoelectrical performance of a single AMR sensor. a, Schematic illustration of the Wheatstone-bridge-type AMR sensor element. The magnetization direction is 45° to all the AMR strips. **b**, Typical response curve of the AMR sensors on Si substrate that shows a linear response with the sweeping magnetic field in the vicinity of zero field condition. The bias voltage is 1 V.

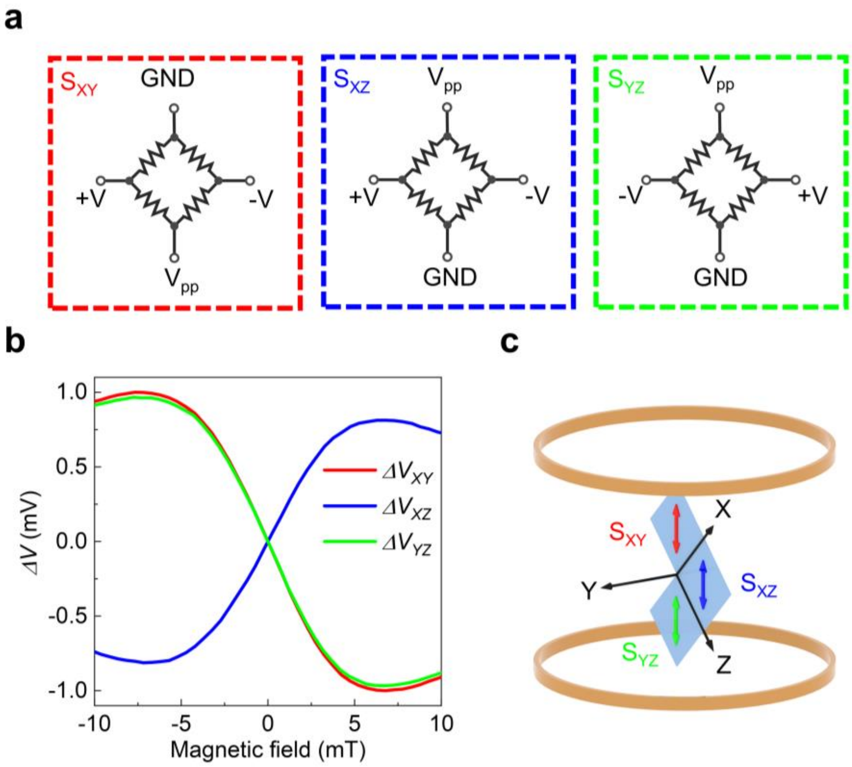

Supplementary Figure 6 | Wheatstone bridge configurations and voltage response of a single pixel in the planar state. a, Illustrations of the Wheatstone bridge configurations for S_{XY}, S_{XZ} and S_{YZ}. S_{XY} and S_{YZ} are electrically equivalent, and S_{XZ} has a reversed configuration compared to the other two. **b**, Typical response of a single pixel in the planar state. Note the response curve of S_{XZ} is flipped due to the bridge configuration as shown in **a**. **c**, Illustration of the sensor orientation in the Helmholtz coils during the field sweeping. The magnetic field is swept along the sensitivity directions for all the three sensors.

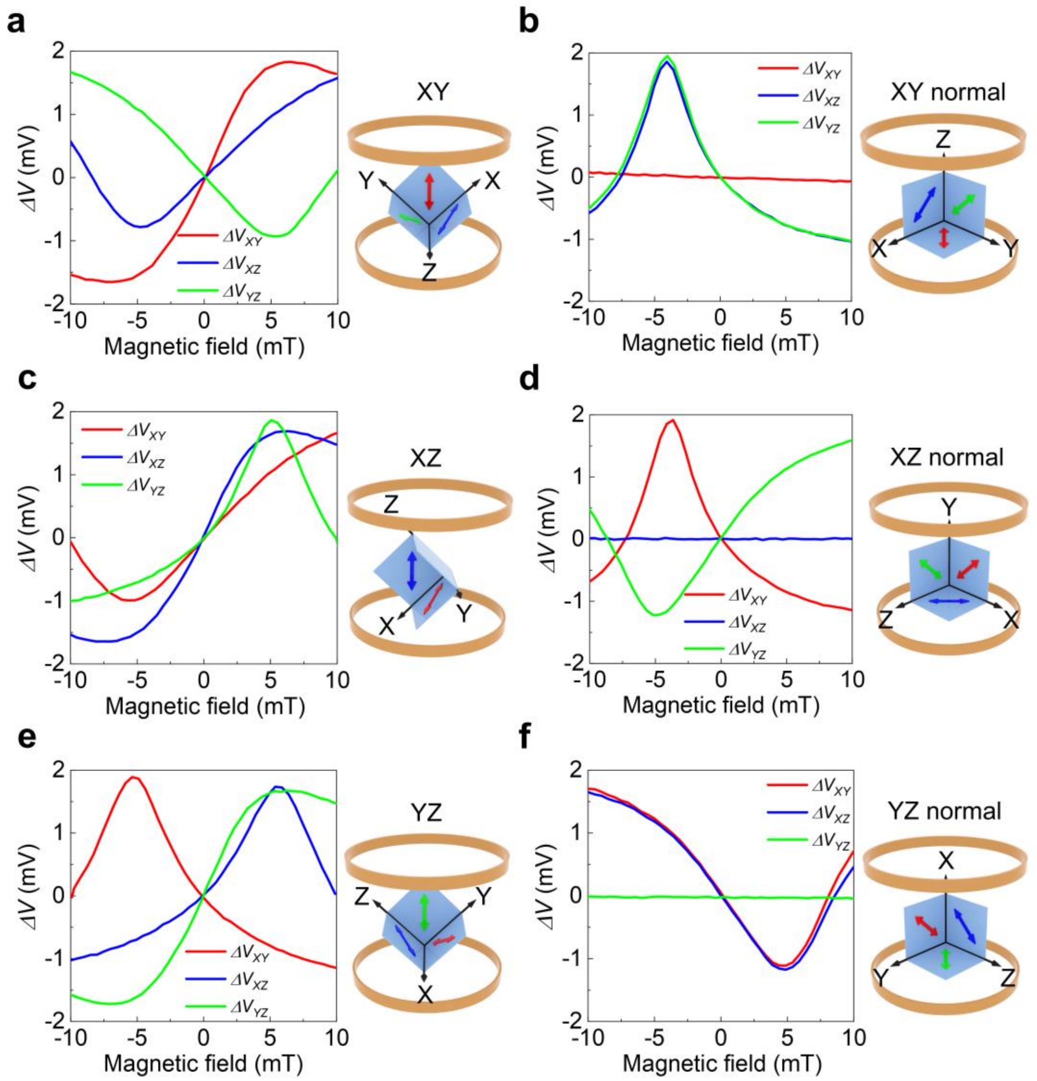

Supplementary Figure 7 | Typical response curves of a single pixel for field swept along different directions. a, Magnetic field direction is along the S_{XY} sensitivity direction. **b**, Magnetic field direction is perpendicular to the XY sensing plane. **c**, Magnetic field direction is along the S_{XZ} sensitivity direction. **d**, Magnetic field direction is perpendicular to the XZ sensing plane. **e**, Magnetic field direction is along the S_{YZ} sensitivity direction. **f**, Magnetic field direction is perpendicular to the YZ sensing plane. The orientations of the sensors and their sensitivity directions in the magnetic field are schematically illustrated respectively.

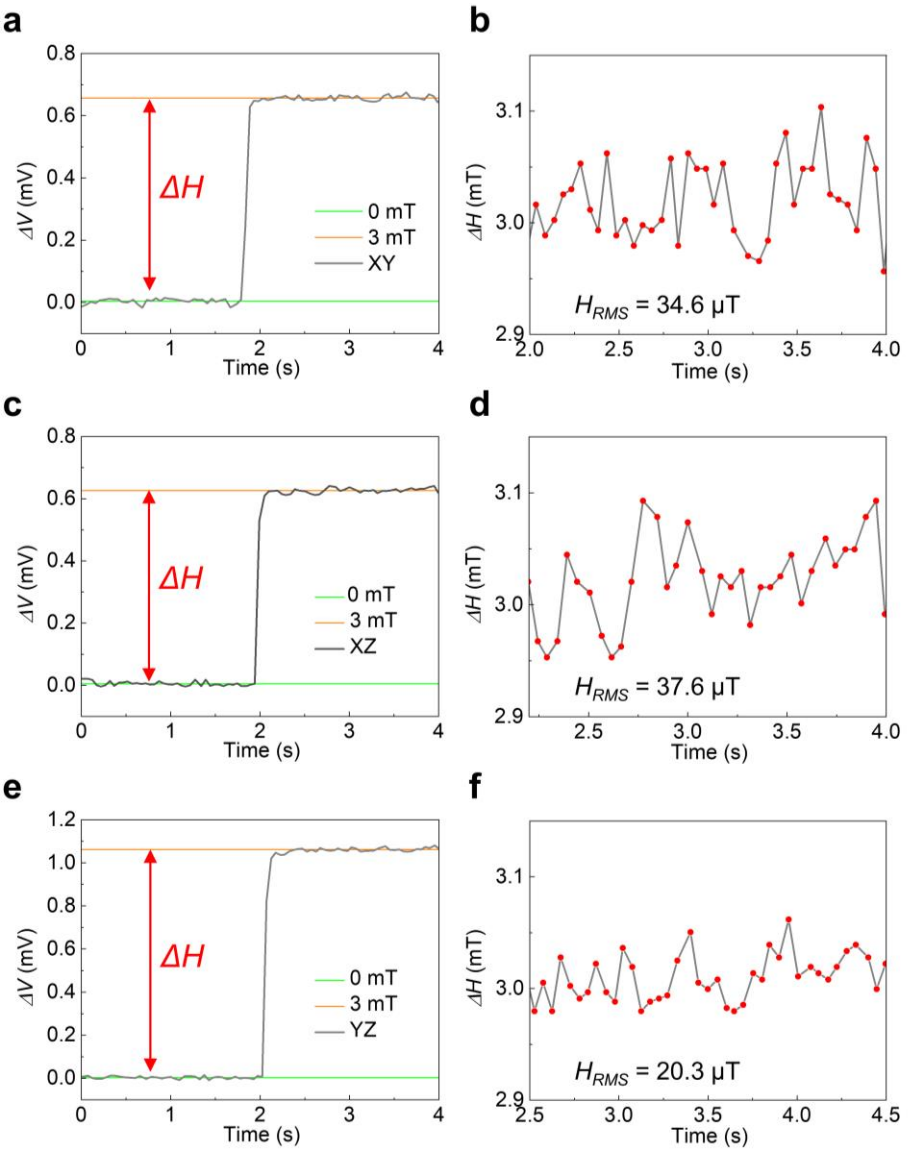

Supplementary Figure 8 | Noise analysis for the sensors. a, **c**, **e**, Typical voltage responses of S_{XY}, S_{XZ} and S_{YZ} to a change of magnetic field from 0 to 3 mT, respectively. The output voltage baselines for the fields of 0 and 3 mT are marked with green and red lines. A voltage step is observed when the field is increased. **b**, **d**, **f**, Noise levels for S_{XY}, S_{XZ} and S_{YZ} respectively. The root-mean-square (RMS) for S_{XY}, S_{XZ} and S_{YZ} are 34.6 µT, 37.6 µT and 20.3 µT respectively.

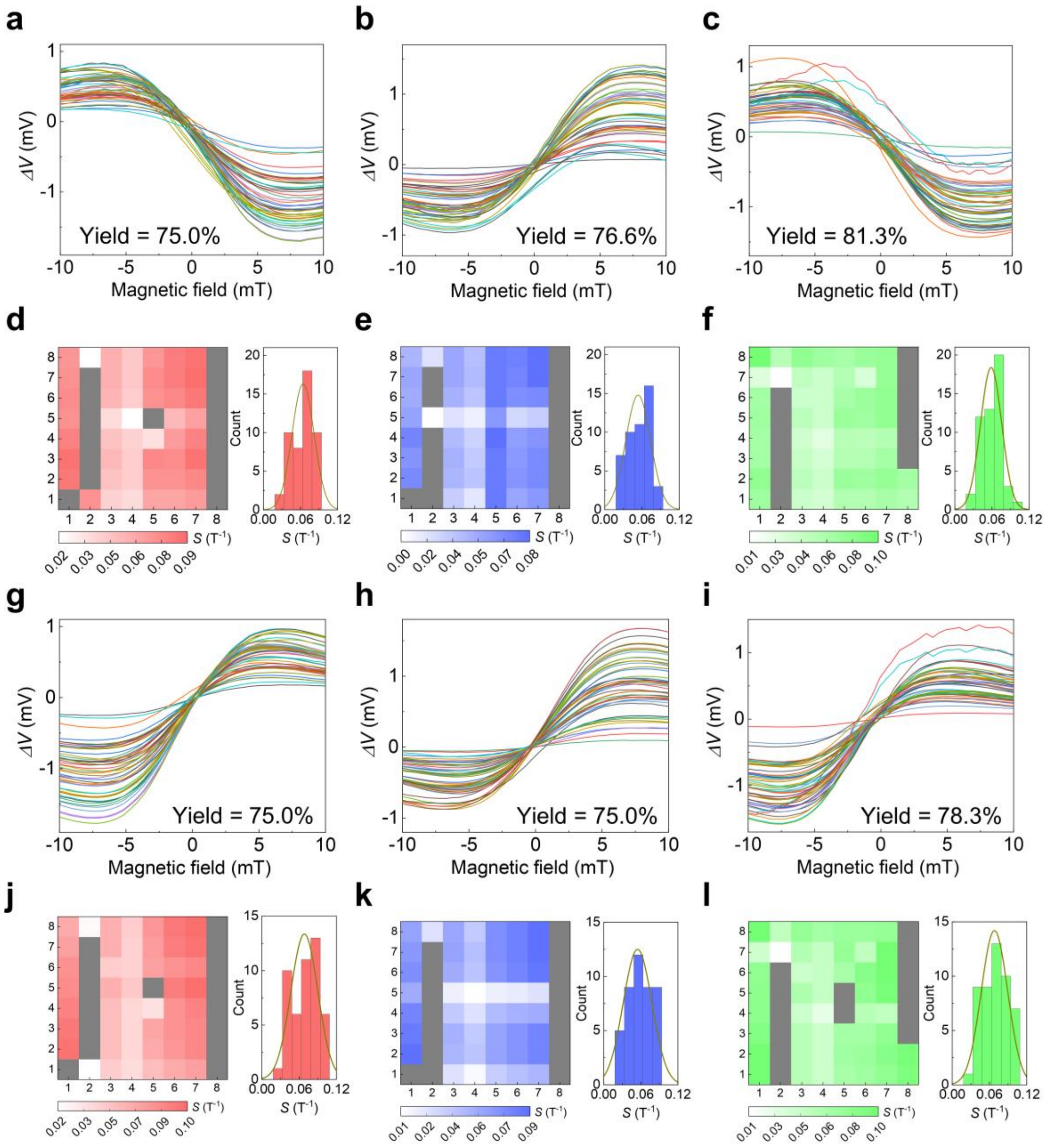

Supplementary Figure 9 | Statistics of the magnetoelectrical performance for the IMOS device before and after the self-folding process. a-**c**, Voltage responses of the working S_{XY}, S_{XZ} and S_{YZ} sensors for a typical 8 × 8 array tested before the self-folding process. The yields for each plane are marked correspondingly. **d**-**f**, Spatial and statistical distribution of the sensitivities before self-folding for the S_{XY}, S_{XZ} and S_{YZ} sensor arrays. The dysfunctional pixels are marked gray. The average sensitivities for the S_{XY}, S_{XZ} and S_{YZ} sensor arrays before folding are (0.064 ± 0.018) T^{-1}, (0.054 ± 0.020) T^{-1}, and (0.059 ± 0.017) T^{-1}, respectively. **g**-**i**, Voltage responses of the working S_{XY}, S_{XZ} and S_{YZ} sensors for the same 8 × 8 array tested after the self-folding process. The yields for each plane are also marked. **j**-**l**, Spatial and statistical distribution of the sensitivities in the folded state for the S_{XY}, S_{XZ} and S_{YZ} sensor arrays. The dysfunctional pixels are marked gray. The average sensitivities for S_{XY}, S_{XZ} and S_{YZ} sensor arrays in the folded state are (0.068 ± 0.022) T^{-1}, (0.055 ± 0.023) T^{-1}, and (0.068 ± 0.021) T^{-1}, respectively. Therefore, the sensitivity and the yield of the sensors are not influenced by the self-folding process.

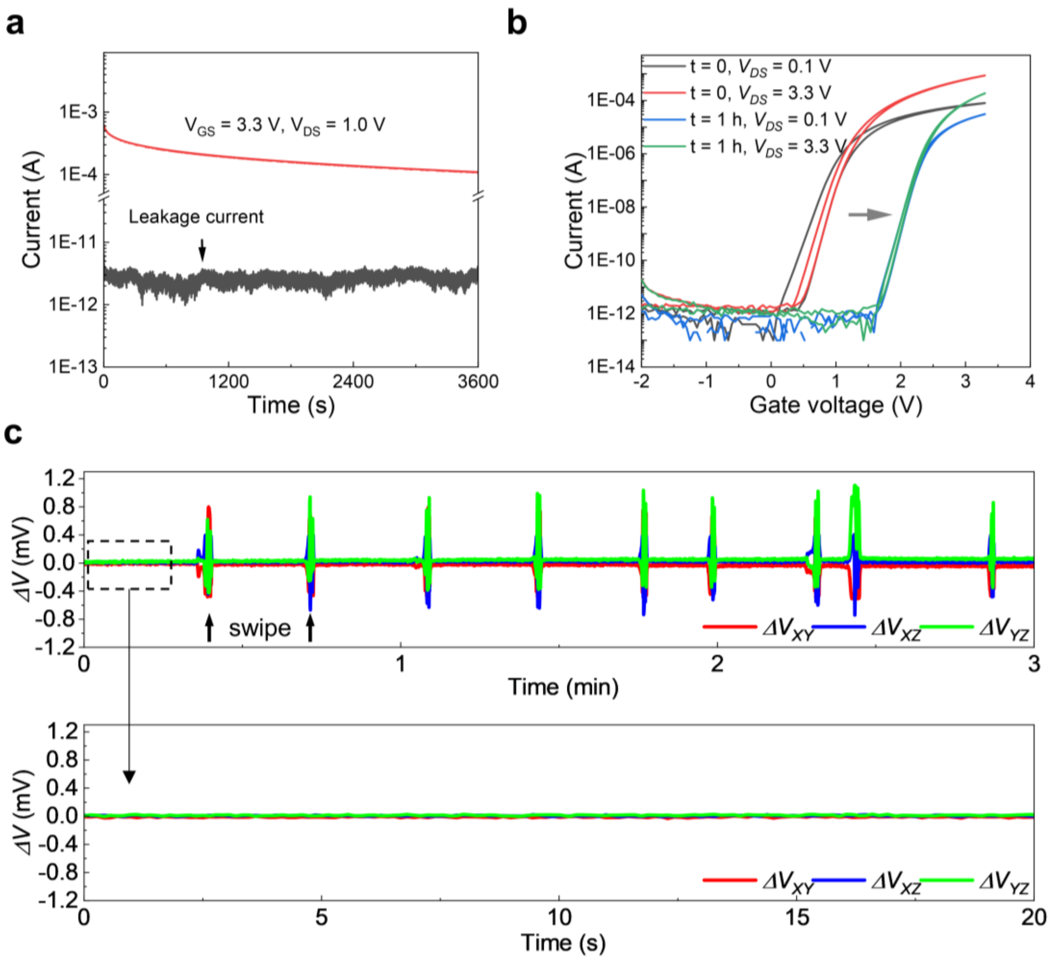

Supplementary Figure 10 | Operational stabilities of the a-IGZO TFTs and the IMOS sensors. a, **b**, Constant-voltage-bias-stress test for the a-IGZO TFTs. **a**, Drain current and gate leakage current change as a function of stress time. Over a stress duration of 3600 s with V_{GS} = 3.3 V, V_{DS} = 1.0 V, the drain current drops within one order of magnitude, and the leakage current dose not increase. **b**, Transfer curves before and after the constant-voltage-bias-stress test over a duration of 3600 s. The constant-voltage-bias stressing results in a positive transfer curve shift and a small decrease of the on-current. **c**, Dynamic stability of the output signal for a typical sensor pixel in the IMOS device. During a continuous recording over 3 min, the baselines for all the three subpixels are stable with very small signal drifting, indicating that the sensors are with high operational stability.

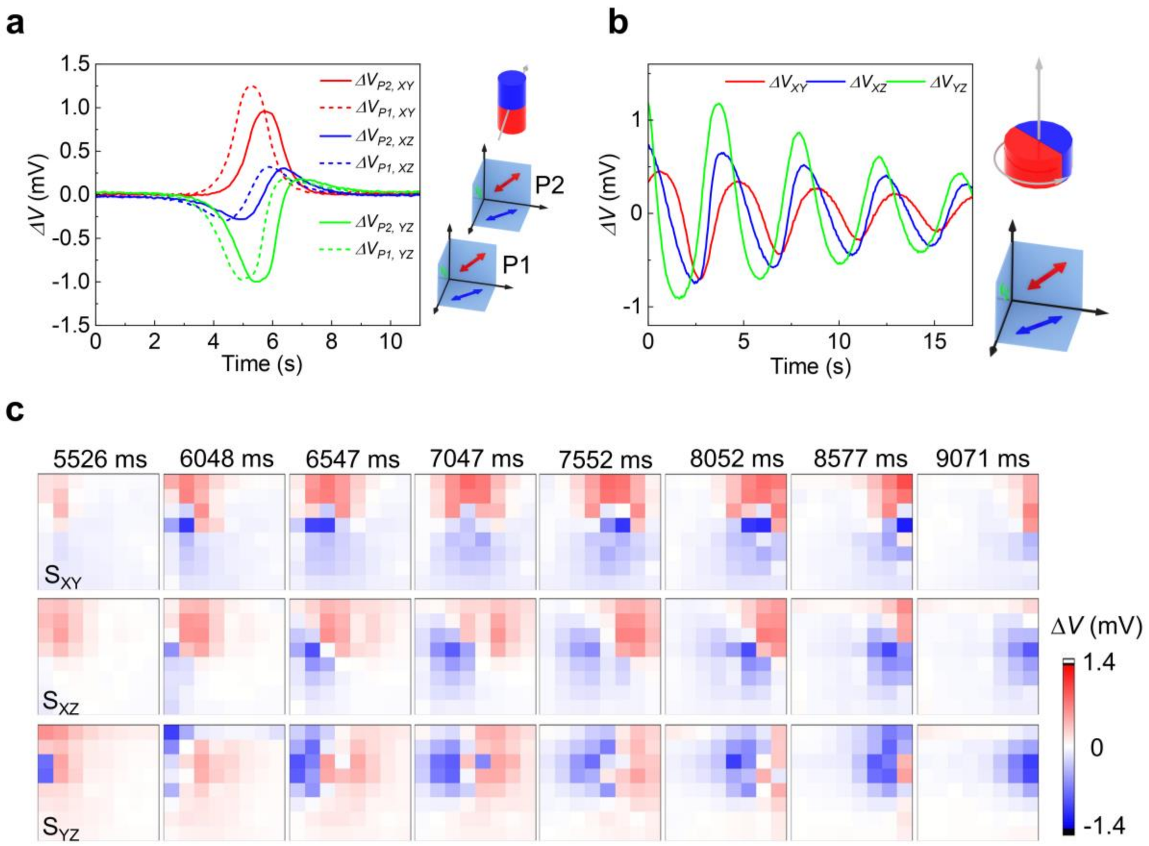

Supplementary Figure 11 | Dynamic tracking of magnetic objects. a, Dynamic voltage response to a linear motion of a NdFeB permanent magnet monitored by two adjacent pixels. A time delay of the response for the two pixels is observed. **b**, Dynamic tracking of the rotation and departure for the NdFeB permanent magnet by a single pixel. The output voltages for S_{XY}, S_{XZ} and S_{YZ} change periodically with decreasing magnitudes. The relative positions of the magnets to the sensor pixels and the magnetization directions of the magnets are schematically illustrated. **c**, Dynamic tracking of a typical linear motion of a NdFeB permanent magnet by the 3D magnetic sensor array. The snapshots of the voltage response for S_{XY}, S_{XZ} and S_{YZ} are plotted respectively.

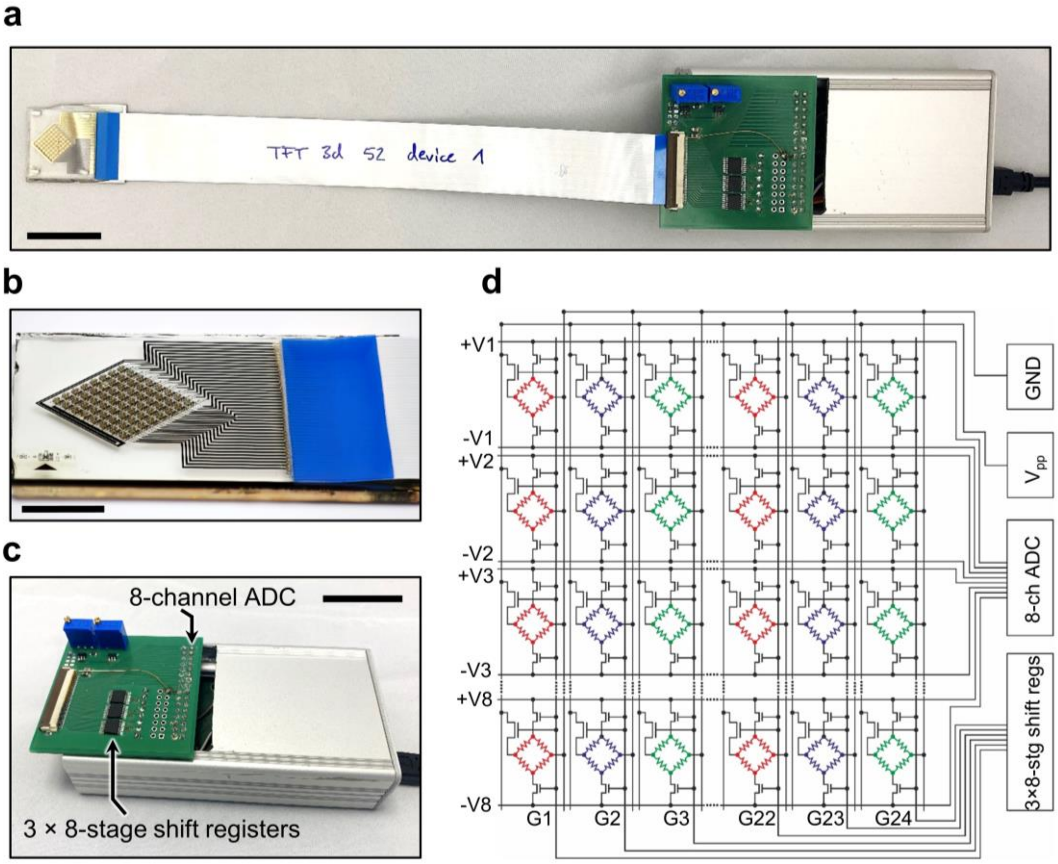

Supplementary Figure 12 | The IMOS device and test circuit. a, Image of an encapsulated IMOS device connected to the test PCB circuit. Scale bar, 25 mm. **b**, Image of an IMOS device bonded to FPC cable. Scale bar, 5 mm. **c**, Image of a test PCB circuit box. The shift registers and the pins for ADC are marked. Scale bar, 25 mm. **d**, Circuit diagram of an IMOS sensor array with connect pins.

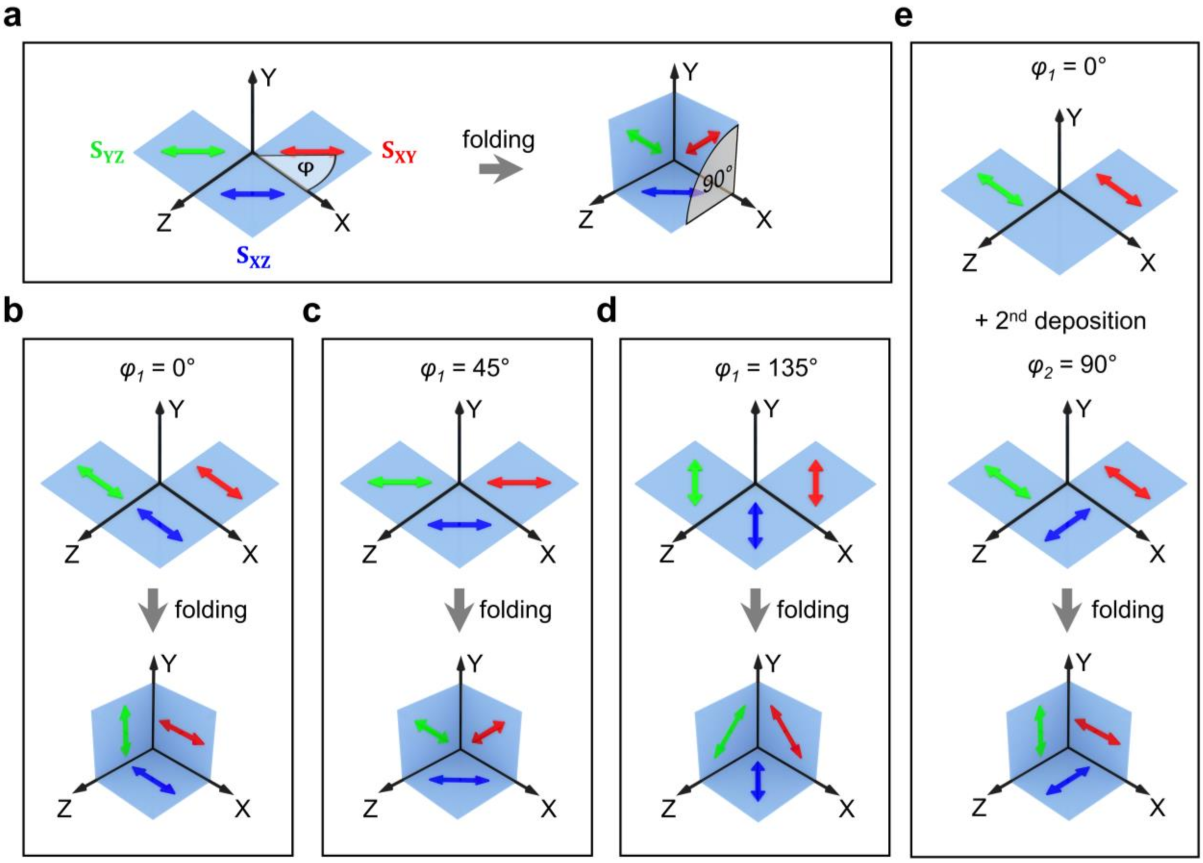

Supplementary Figure 13 | Sensitivity directions before and after the self-folding process. a, The sensitivity direction is defined as the angle φ with respect to the X-axis. For the folded-up state an orthogonal alignment of all the planes can be assumed. For **b-d**, a one-step deposition and magnetization of the sensors is considered. **b**, Case 1: $\varphi_1 = 0°$ **c**, Case 2: $\varphi_1 = 45°$. **d**, Case 3: $\varphi_1 = 135°$. **e,** Case 4: A sequential sensor fabrication process is considered. Two subsequent sensor deposition and magnetization steps with perpendicular sensitivity directions ($\varphi_1 = 0°$, $\varphi_2 = 90°$) result in orthogonal sensitivity direction alignment in the folded-up state.

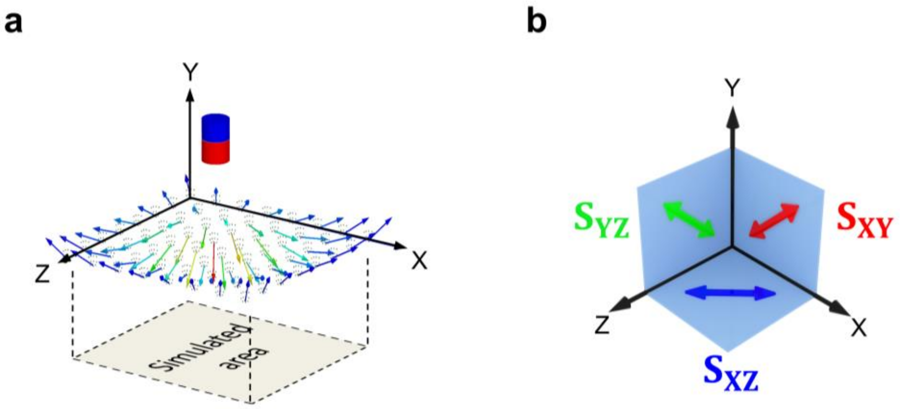

Supplementary Figure 14 | Magnetic vector field simulation. a, Vector field of a rod magnet in a plane (XZ) at a distance of 2 mm to the magnet. **b**, Orthogonal base and orientation of the sensitivity directions $\mathbf{S_{XY}}$ (red arrow), $\mathbf{S_{XZ}}$ (blue arrow) and $\mathbf{S_{YZ}}$ (green arrow).

Supplementary Tables

Supplementary Table 1 | Normalized sensitivity directions for the planar and folded states.

Case	Planar sensitivity directions			Folded sensitivity directions		
	XY	XZ	YZ	XY	XZ	YZ
1 ($\varphi_1 = 0°$):	(1, 0, 0)	(1, 0, 0)	(1, 0, 0)	(1, 0, 0)	(1, 0, 0)	(0, -1, 0)
2 ($\varphi_1 = 45°$):	$\left(\frac{1}{\sqrt{2}}, 0, -\frac{1}{\sqrt{2}}\right)$	$\left(\frac{1}{\sqrt{2}}, 0, -\frac{1}{\sqrt{2}}\right)$	$\left(\frac{1}{\sqrt{2}}, 0, -\frac{1}{\sqrt{2}}\right)$	$\left(\frac{1}{\sqrt{2}}, \frac{1}{\sqrt{2}}, 0\right)$	$\left(\frac{1}{\sqrt{2}}, 0, -\frac{1}{\sqrt{2}}\right)$	$\left(0, -\frac{1}{\sqrt{2}}, -\frac{1}{\sqrt{2}}\right)$
3 ($\varphi_1 = 135°$):	$\left(-\frac{1}{\sqrt{2}}, 0, -\frac{1}{\sqrt{2}}\right)$	$\left(-\frac{1}{\sqrt{2}}, 0, -\frac{1}{\sqrt{2}}\right)$	$\left(-\frac{1}{\sqrt{2}}, 0, -\frac{1}{\sqrt{2}}\right)$	$\left(-\frac{1}{\sqrt{2}}, \frac{1}{\sqrt{2}}, 0\right)$	$\left(-\frac{1}{\sqrt{2}}, 0, -\frac{1}{\sqrt{2}}\right)$	$\left(0, \frac{1}{\sqrt{2}}, -\frac{1}{\sqrt{2}}\right)$
4 ($\varphi_1 = 0°$; $\varphi_2 = 90°$):	(1, 0, 0)	(0, 0, 1)	(1, 0, 0)	(1, 0, 0)	(0, 0, 1)	(0, -1, 0)

Supplementary Notes

Supplementary Note 1 | Rearrangement of sensitivity directions by the self-folding process

We define the orientation of the sensitivity direction for the sensors in the planar state by its angle φ with respect to the X-axis (Supplementary Fig. 13a). The sensitivity direction for the sensors in the planar state can be written as the vector defined by

$$\mathbf{S} = \begin{pmatrix} \cos\varphi \\ 0 \\ -\sin\varphi \end{pmatrix} \qquad (1)$$

If we consider the rearrangement of the sensors deposited on the XY and YZ planes upon folding, the predefined directions are effectively rotated about the X-axis and Z-axis, respectively. This rotation can be mathematically described by a rotation matrix from which the final sensitivity directions can be obtained. In our origami design, the folding regime is fixed to a 90° folding angle, resulting in the orthogonal orientation of all the planes. For an arbitrary sensitivity direction φ (0 - 360°), set in the planar state for all the sensors in parallel, the sensitivity directions for the sensors on the XY and YZ planes in the folded-up state can be written as:

$$\mathbf{S_{XY}} = \begin{pmatrix} 1 & 0 & 0 \\ 0 & 0 & -1 \\ 0 & 1 & 0 \end{pmatrix} \begin{pmatrix} \cos\varphi \\ 0 \\ -\sin\varphi \end{pmatrix} = \begin{pmatrix} \cos\varphi \\ \sin\varphi \\ 0 \end{pmatrix} \qquad (2)$$

$$\mathbf{S_{YZ}} = \begin{pmatrix} 0 & 1 & 0 \\ -1 & 0 & 0 \\ 0 & 0 & 1 \end{pmatrix} \begin{pmatrix} \cos\varphi \\ 0 \\ -\sin\varphi \end{pmatrix} = \begin{pmatrix} 0 \\ -\cos\varphi \\ -\sin\varphi \end{pmatrix} \qquad (3)$$

Several cases with specific initial planar sensitivity directions are sketched in Supplementary Fig. 13b-d to demonstrate the rearrangement of the sensitivity directions by the self-folding micro-origami process. This corresponds to a one-step deposition and magnetization of all the sensors which have parallel sensitivity directions. For this reason, the angle is written as φ_1. The presented cases show that for a single sensitivity direction in the planar state only two orthogonal directions can be obtained in the folded-up state. For φ_1 = 135°, a quasi-orthogonal base can be obtained, which can still be used to measure all the magnetic field components. A sensor configuration resulting in three orthogonal sensitivity directions can be fabricated by two-step depositions of the sensors. For instance, sensors on the XY and YZ planes can be first deposited with φ_1 = 0°, and sensor on the XZ plane can be deposited by setting φ_2 = 90° (Supplementary Fig. 13e). The final folding process will bring all the sensors in a truly orthogonal alignment. The sensitivity directions for all the four cases have been calculated based on (2) and (3) and are summarized in Supplementary Tab. 1.

Note that for case 1 and case 4, the defined sensitivty directions would require a rotation of the planar sensor layout as the magnetization direction should have a 45° angle to the stripe elements in the AMR Wheatstone bridge.

Supplementary Note 2 | Finite elements simulation results

The magnetic stray field, originating from a small permanent magnet is simulated in the finite elements simulation (FEM) software Analysis System (ANSYS Academics 17.2). Results are exported for a regular grid of points on a square shaped planar area with a distance of 2 mm to the magnet. The total, exported simulated area is 8.8 × 8.8 mm^2 and the grid spacing/pitch is 0.1 mm in X- and Z- direction, respectively, while to eliminate edge effects vacuum box surrounding the model was 20 × 20 × 20 mm^3. Therefore, the simulation map spatial resolution is higher than experimental maps obtained from the IMOS device. The magnetic vector field, typical for a rod magnet with axial magnetization, reveals a strong out-of-plane component (Y-direction in Supplementary Fig. 11a) along the magnet's magnetization direction. As the magnetic flux lines are always closed, the magnetic vector rotates towards the antiparallel direction at the edges of the simulated plane. From the magnetic vector field in the observed plane (Supplementary Fig. 11a), the three orthogonal vector components X, Y and Z are extracted at each point. The three magnetic vector components are the projections of the magnetic field vector on the axes of an orthogonal base (Supplementary Fig. 11b). From these components the actual projection of the magnetic field vector on the three orthogonal planes XY, XZ and YZ can be obtained.

$$\mathbf{XY} = \begin{pmatrix} X \\ Y \end{pmatrix};\ \mathbf{XZ} = \begin{pmatrix} X \\ Z \end{pmatrix};\ \mathbf{YZ} = \begin{pmatrix} Y \\ Z \end{pmatrix} \quad (1)$$

Here, $\mathbf{XY}$, $\mathbf{XZ}$ and $\mathbf{YZ}$ are the projections of the magnetic field vector $\mathbf{H}$ on the three orthogonal planes XY, XZ and YZ, obtained from the vector components X, Y and Z. Projecting $\mathbf{XY}$, $\mathbf{XZ}$ and $\mathbf{YZ}$ on the corresponding magnetic sensor sensitivity axes

$$\mathbf{S_{XY}} = \begin{pmatrix} -1 \\ -1 \end{pmatrix};\ \mathbf{S_{XZ}} = \begin{pmatrix} 1 \\ -1 \end{pmatrix};\ \mathbf{S_{YZ}} = \begin{pmatrix} 1 \\ 1 \end{pmatrix} \quad (2)$$

gives the expected signal responses of the 3D sensor pixel

$$V_{XY} = \frac{\mathbf{S_{XY}} \cdot \mathbf{XY}}{|\mathbf{XY}|};\ V_{XZ} = \frac{\mathbf{S_{XZ}} \cdot \mathbf{XZ}}{|\mathbf{XZ}|};\ V_{YZ} = \frac{\mathbf{S_{YZ}} \cdot \mathbf{YZ}}{|\mathbf{YZ}|} \quad (3)$$

Note that the sensitivity vectors are defined by the magnetization direction in the planar state and the subsequent self-folding, resulting in a rearrangement of the sensors and its sensitivity vectors.
