# Supplementary material for: A new dimension for magnetosensitive e-skins: active matrix integrated micro-origami sensor arrays
Source: Nat Commun. 2022 Apr 19;13:2121. doi: 10.1038/s41467-022-29802-7 (PMC9018910; doi:10.1038/s41467-022-29802-7)
Supplement: Supplementary file 3 — Description of Additional Supplementary Information [file 41467_2022_29802_MOESM3_ESM.pdf]

Description of Additional Supplementary Information

Title: Supplementary Movie 1

Description: Real time 3D mapping of magnetic stripe.

Title: Supplementary Movie 2

Description: Real time 3D mapping of small permanent magnet.
